# The Point of No Return? Impediments to Return to Work for Injured Migrant Agricultural Workers in Two Canadian Provinces

**DOI:** 10.1177/10482911251314149

**Published:** 2025-02-21

**Authors:** Stephanie Mayell, Janet McLaughlin, Jenna Hennebry, Guillermo Ventura Sanchez, Pankil Goswami, Jill Hanley

**Affiliations:** 1 Department of Anthropology, 7938 University of Toronto, ON, Canada; 2 International Migration Research Centre, Wilfrid Laurier University, Waterloo, ON, Canada; 3 Department of Health Studies, Wilfrid Laurier University, Brantford, ON, Canada; 4 Department of Sociology and Anthropology, 5618 Concordia University, Montreal, QC, Canada; 5 School of Social Work, 5620 McGill University, Montreal, QC, Canada

**Keywords:** agricultural workers, migrant workers, occupational injury, occupational illness, workers’ compensation, return-to-work, Canada, Temporary Foreign Worker Program

## Abstract

Migrant agricultural workers employed through Canada's Temporary Foreign Worker Program face serious occupational health and safety hazards, with compounded difficulties in accessing workers’ compensation (WC) if they are sick or injured by the job. Little is known, however, about their ability to return to work (RTW) upon recovery—a fundamental right included in the conception of WC, but complicated by their restrictive work permits and precarious immigration status. Based on interviews with injured migrant workers in two Canadian provinces (Quebec and Ontario), our research suggests that workers’ RTW process is anything but straightforward. This article highlights three key issues—pressure to return to work prematurely, communication and bureaucratic challenges with WC agencies, and impacts of injury/illness and failure to return to work on workers’ long-term well-being. Consequences and opportunities for reform are discussed.

## Introduction

In 2023, more than 70,000 migrant agricultural workers were employed under Canada's Temporary Foreign Worker Program (TFWP); approximately 30,000 worked in Ontario and another 23,000 in Quebec.^1^ Workers are hired from dozens of countries, but the majority come from Mexico, Guatemala, and Jamaica, thanks to agreements with those governments.^2^ The structures of Canada's migrant worker programs specific to the agricultural sector create unique migration realities^3^ and significant challenges for migrant workers’ health and the protection of their rights.^4–8^ Migrant agricultural workers’ temporary status and work permits in Canada are tied to a single employer (i.e., they are closed work permits),^9^ who can terminate their employment (and thereby their status) for any reason (e.g., lack of work available, worker refusing to work excessive overtime, and worker unable to work due to health reasons), while workers have no direct grievance process. In Canada, migrant agricultural workers live in employer-provided accommodations, but there are no explicit national housing standards to ensure healthy, safe, and decent housing, and workers’ accommodations are often substandard and linked to poor physical and mental health outcomes.^10^

Agriculture consistently ranks among Canada's most dangerous occupations for fatal and nonfatal work-related injuries.^11–13^ Common health issues include injuries; pain in the back, neck, knee, shoulders, hands, or feet; conjunctivitis; corneal foreign bodies and abrasions; pterygia (an eye condition); dermatitis; folliculitis; tinea (ringworm infection); depression; anxiety; poor sleep patterns; and sexually transmitted infections.^14^ There is also a higher rate of cancer cases among agricultural workers, especially among those who apply pesticides or are present when they are being used.^15–17^

The health hazards migrant agricultural workers face are exacerbated by their precarious immigration status and closed work permits, which make them practically unable to refuse dangerous or unhealthy work, a right in all Canadian health and safety laws.^18–21^ Furthermore, in most provinces, including Ontario and Quebec, agricultural workers are excluded from numerous labor rights and employment standards. To begin, migrant agricultural workers are excluded from several key labor standards under Ontario's *Employment Standards Act* (ESA) and Quebec's *Loi sur les normes du travail* (LNT), including lesser protections in terms of hours of work, rest periods, statutory holidays, and overtime,^22,23^ making them especially vulnerable to exploitation and injury.^24–26^ In both provinces, the labor relations laws include provisions specific to agricultural workers, effectively limiting their ability to join a union and/or bargain collectively.^21,27–29^

In terms of occupational health and safety (OHS), while agricultural workers are covered by Ontario's *Occupational Health and Safety Act* (OHSA)^30^ and Quebec's *Loi sur la santé et la sécurité au travail* (LSST),^31^ in practice their access is highly constrained. In general, workers are worried about the consequences of refusing to work in unhealthy or dangerous conditions, fearing repatriation or not being recalled the next season.^32–35^ In many cases, workers wait until they get back to their home countries to receive surgeries or check-ups.^36^

These factors all intersect with migrant workers’ underlying vulnerabilities and set the stage for numerous complications in the workers’ compensation (WC) and return-to-work (RTW) processes. This paper explores these issues, focusing on the factors that influence the RTW process in Quebec and Ontario, Canada's two largest and most populous provinces.

## Policy Context (Return-to-Work)

Injured or ill migrant agricultural workers in Ontario^37^ and Quebec^38^ are entitled to provincial health insurance, yet they experience many barriers to accessing health services.^39,40^ For example, employers are responsible for facilitating workers’ applications for health cards and distributing them at the beginning of workers’ contracts, making migrant workers dependent on employers to realize their health rights.^41,42^ This is made worse by the common practice of employers holding workers’ health cards for “safety” reasons. Sometimes employers fail to facilitate applications for the health card, or they do so later into the season when health issues may already have emerged.^7^

Even if they receive their card, migrant workers often depend on employers for transportation to see health-care providers. Workers who want to access health-care independently encounter several practical barriers. These include long hours of work and limited clinic hours in rural areas; lack of cultural sensitivity among health-care providers, transportation, and information about and integration into the local health-care system; confidentiality concerns due to reliance on employers or supervisors; and language and literacy barriers. Most significantly, structural barriers related to federal laws about immigration and provincial ones about health, combined with a lack of government monitoring of work conditions, generate significant power imbalances that make rights and entitlements difficult to use. Doing so largely depends on negotiating access through the employer who influences workers’ jobs, their housing, and their ability to stay in and/or return to Canada.^43–46^ Moreover, seriously injured and sick workers are often repatriated before receiving health-care or compensation.^47^ These intersecting challenges mean migrant agricultural workers in Canada are structurally vulnerable to poor health outcomes.^48–51^

After a job-related injury or illness, workers, employers, and health-care providers are each supposed to fill in WC forms for a claim to be made. Injured and sick migrant workers face numerous barriers to accessing this coverage, including structural and logistical challenges and insufficient education about WC, on top of language and cultural differences.^8,21,52^ Additionally, health professionals often do not know how to navigate the immigration restrictions of the closed work permit and may not even know that migrant workers are eligible for compensation.^15^ Employers also may be economically motivated to not fill in their claim forms. These combined reasons make under-reporting common.^40^

When a claim is made, however, the Ontario and Quebec WC agencies prioritize an early RTW process. Both emphasize minimizing time off for recovery and focus on facilitating a worker's return to suitable, healthy, and safe work.

The Ontario WC system is administered by the Workplace Safety and Insurance Board (WSIB). Benefits and services in case of occupational injury or illness are outlined in the *Workplace Safety and Insurance Act* (WSIA).^53^ In Quebec, the WC administration is the responsibility of the *Commission des normes du travail, de l’équité salariale, de la santé et de la sécurité au travail* (CNESST—Commission of Labour Standards, Pay Equity, Occupational Health and Safety). The WC procedure is governed by the *Act Respecting Industrial Accidents and Occupational Diseases*.^54^ Both use a legal and policy framework mandating the responsibilities of employers and workers in the RTW process and provide guidelines and regulations to ensure that it is consistent and fair.^55–57^

The health and safety of people returning to work after an injury or illness is a key component of both provinces’ RTW policies. They say employers must make any necessary adjustments to the workplace to accommodate the returning worker's needs and ensure a healthy and safe working environment. This means employers are expected to provide modified or alternative duties (e.g., light duties) to accommodate the worker's abilities, and workers are encouraged to actively participate in the RTW planning. Both jurisdictions have mechanisms to coordinate and support the RTW process to ensure it is managed effectively and potential barriers are addressed. Personnel include RTW coordinators, health-care providers, and rehabilitation specialists. Both the worker and the employer are legally obliged to cooperate in the early RTW process.

In the experience rating system both agencies use, employers are charged levies depending on the number and duration of WC claims made. This makes them economically motivated to offer modified duties that let WC claimants keep working and stop getting compensation. Once a worker's WC claim is accepted, they must agree to offers of modified work; theoretically, these alternate duties should not harm workers’ health.^58^ However, physicians are not called upon to approve the proposed task(s). Instead, injured workers are generally required to visit a WSIB or CNESST assessment center. In both provinces, only workers who are citizens or permanent residents, who are no longer able to work in their former field, are eligible for retraining meant to prepare them for employment in a different field suited to their new needs/abilities.

The Ontario and Quebec RTW procedures, policies, and services are meant to serve all workers equally; however, inequities within Canada's health and safety and WC systems present serious challenges for vulnerable resident workers, including women, older individuals, and racialized workers. Compared to their Canadian-born or permanent resident counterparts, immigrant workers in particular face significant occupational health disparities and increased risk of occupational injury or illness.^59^ Lack of English proficiency makes many of these workers vulnerable to abuse and misunderstanding by health-care providers, employers, and WC adjudicators.^60^ Injured immigrant workers who file WC claims experience myriad challenges navigating WC systems due to communication barriers and lack of familiarity with the procedures, and they are less likely to return to work following an occupational injury or disease.^61^ There are similar challenges in both provinces related to language barriers and cultural factors for immigrant workers navigating the RTW process.^15,62,63^

These challenges are further compounded for nonresident migrant workers. Prior research with migrant workers in Canada found the intersection of workers’ temporary migration and employment status presents unique barriers for RTW.^21,52,64,65^ Many injured and sick migrant workers are repatriated prematurely rather than accommodated so they can return to work,^47^ and those who return home before the conclusion of their WC process do not typically benefit from re-employment, retraining, and/or reintegration into any labor market. If a worker's injury or illness persists, they normally are excluded from future employment under Canada's TFWP, as they cannot pass the annual medical examination or perform the intensive manual labor required in most agricultural positions. Employers may also be reluctant to rehire previously injured or sick workers. Consequently, injured workers often experience long-term unemployment and severe economic hardship. Meanwhile, bureaucrats in Canada and their home countries blame each other for not taking responsibility for the health of temporary workers.^15^ Our research sought to determine the scope of these problems for migrant workers in Ontario and Quebec and to identify effective policy solutions to improve RTW outcomes.

## Methodology

This study was part of a larger national project exploring policy and practice related to occupational injury and illness prevention and rehabilitation in Canada among vulnerable workers.^a^ Specifically, our research aimed to accomplish two main objectives: to investigate the RTW barriers and facilitators for sick and injured migrant workers across TFWP streams and propose policy solutions to ensure optimal RTW outcomes for migrant workers who are injured or become ill in Ontario and Quebec.

Our methods included an analysis of WC policies and semistructured interviews with 44 migrant (temporary foreign) workers in Ontario and Quebec. Worker interviews lasted approximately 1 hour, focusing on themes including preinjury work conditions, the nature and circumstances of their workplace injury or illness, the aftermath (e.g., employers’ reaction, health consequences, access to health-care), interactions with the WC agency, and the RTW process. Interview questions were designed to solicit demographics and personal background information, details about the occupational injury/illness, if a WC claim was filed, challenges during the claims process, the impact of COVID-19, and factors that influenced the RTW experience, such as precarious housing and repatriation. Our interview guides are available as supplemental material.

Participant characteristics are presented in Table 1. In Ontario, we interviewed 23 injured or sick migrant workers from Jamaica (n = 8), Mexico (n = 7), St. Vincent and the Grenadines (n = 6), Barbados (n = 1), and Guatemala (n = 1). Most participants were male (n = 18). All but one worked in agriculture (n = 22); the other was in the hospitality industry. In Quebec, we interviewed 21 injured or sick workers (female n = 2, male n = 19) from Mexico (n = 6), Guatemala (n = 11), Jamaica (n = 1), Morocco (n = 1), and Cameroon (n = 2). Only six worked directly in primary agriculture. Others worked in connected sectors (livestock n = 8; agricultural machinery n = 1; agricultural landscaping n = 1). The remaining five in Quebec worked in landscaping (n = 2), construction (n = 1), hospitality (n = 1), and welding (n = 1).

**Table 1.** Characteristics of Participants.

Characteristics		Ontario participants (N = 23)	Quebec participants (N = 21)	Total participants (N = 44)
Gender	Male	18	19	37
	Female	5	2	7
Country of origin	Barbados	1	0	1
	Cameroon	0	2	2
	Guatemala	1	11	12
	Jamaica	8	1	9
	Mexico	7	6	13
	Morocco	0	1	1
	St. Vincent and the Grenadines	6	0	6
Occupation	Primary Agriculture	22	6	28
	Livestock	0	8	8
	Agricultural Machinery	0	1	1
	Agricultural Landscaping	0	1	1
	Hospitality	1	1	2
	Landscaping	0	2	2
	Construction	0	1	1
WC claim filed	Yes	20	12	32
	No	3	9	12

Participants were not required to have made a WC claim. In Ontario, 20 had filed claims, 3 had not; in Quebec, 12 had filed claims, and 9 had not. In this paper, we focus primarily on the RTW experiences of injured agricultural workers who filed WC claims in either province (n = 32).

This project was approved by research ethics boards at the participating universities. Interviews were conducted either in-person or virtually via Zoom or WhatsApp video call in English, French, or Spanish. Participants were in Ontario, Quebec, Jamaica, Mexico, and St. Vincent and the Grenadines. All interviews were audio-recorded, transcribed, and translated into English as needed. Transcriptions were de-identified, reviewed, and cleaned for accuracy. Identifying information was stored separately and made available exclusively to the research team.

Multiple team members reviewed the final transcripts and identified common themes. Subsequently, the data was inductively sorted into codes. Coded data was organized in spreadsheets. All data was encrypted and stored on a secure university server. All participants were assigned pseudonyms, which are used in this article.

## Results

We found that injured or sick migrant workers in both provinces reported several challenges in accessing RTW services and recovering both their health and employment, with strong parallels in Ontario and Quebec. We have highlighted three of the most common themes that emerged consistently: (1) pressure to return to work prematurely; (2) communication and bureaucratic challenges with WC agencies; and (3) long-term impacts of failed RTW on workers.

### Pressure to Return to Work Prematurely

In the early days following an occupational injury or illness, employers legally must file a WC report^66,67^ and make reasonable efforts to modify the work and/or workplace to enable the injured worker to perform their job or offer alternative suitable work that will not exacerbate their injury or illness. Despite this, many injured workers reported feeling pressured to return to work too soon and to perform work that did not feel healthy or safe.

For example, a Jamaican worker named Andre had a serious back injury while working at an Ontario vegetable farm. Although he did file a WC claim, Andre explained that in the days and weeks following his injury, his employer focused solely on getting him back to work to maintain production, not on his health: “My employer wants me to go back to work but I’m injured. And if I go back to work then now it would be a serious condition on me*.*” Reticent to speak up and advocate for his need for health care and light duties, Andre ultimately decided to abandon his job.

Eduardo, a Mexican worker who suffered a repetitive strain injury from packing boxes for up to 17 h per day, had a similar experience: “The company only cares that you work, they don’t really care what you’re feeling, what your sufferings are, what hurts you. You are only important if you are working.” He was reluctant to take any time off work to rest and recuperate, because he would not be paid, and his family relied on his income:

I could no longer afford to rest when I didn’t know if I was going to get paid or what… many [injured] people prefer to work like this because they know that if they are going to rest… well, they’re not going to pay for their day, they’re not going to get paid for their hours… So, most of them… they work to not lose hours.

Despite workers having the right to income replacement for job-related illness or injury, it was common for participants to report loss of income as a major concern. Financial need also inspired Augusto, a Guatemalan worker, to return to work before he was physically ready. He explained:

I was disabled six weeks, of which I only took five… one of the reasons why I returned a week before to work was also because the [WSIB] cheque did not arrive soon and well, I have family in my country, and well… they depend on me, right?

In his case, waiting to get his benefits cheque in the mail prohibited Augusto from caring for his family back home. He felt he had no choice but to return to work before he should have.

Quebec workers also reported employers pressured them to get back to work despite their pain and limited abilities. For example, Macario, a Guatemalan livestock worker, said he was ordered back to work after his injury: “I told them that I was in pain, and I couldn’t do anything, and they wanted me to go to work because I was in charge of giving milk to the calves.” Likewise, another Guatemalan worker, Luis, whose employer delayed his ability to file a WC claim, said:

The most serious accident I had was when… a recently calved cow… jumped very high and kicked me in the knee. And I was limping around the farm, and I thought I wasn’t going to work anymore because my knee swelled up. I thought I wasn’t going to work, but the boss didn’t care about that. He did not take me to the hospital, and I continued working. I was limping for a long time on the farm, and I told the boss to take me to the hospital and he told me he didn’t have time to take me.

Many workers reported that their employers disregarded the compensation agencies’ RTW recommendations. In Ontario, workers told us their employers often agreed to meet their responsibilities in the presence of WC representatives but then failed to provide the modified work/light duties to which they had agreed. Hugo, a worker from Guatemala, explained:

They [the boss] had promised to give me light work… I was not going to be lifting weight, I was not going to walk all day or be standing all day. But when I return, [the employer] tell me that they do not know where to put me to work, that they do not have soft work there.

Garfield, a worker from Jamaica injured working at a vegetable farm, told us his employer also failed to give him light duties, while telling the compensation agency that they had done so. Trying to perform the strenuous duties assigned made things worse:

[It] was documented in the file that I am doing light duty, which wasn’t so. I went back out in the field…that day I actually was partially paralyzed by midday, I couldn’t walk, couldn’t nothing. I had to be lifted to the boss and they did not take me to the doctor.

Although light or modified work was offered in a few Quebec cases, the overwhelming majority of employers did not do so. Instead, injured workers were either fired or pressured to continue working at their regular tasks. For example, Ruben from Guatemala explained that his employer's lack of respect for his need to work lighter tasks led to his decision to leave the job:

It is supposed [to be] if you are sick, they are going to rest you. But we did not rest, they put me to work… I told the doctor to please give me a document so I could rest, I told him they always put me to work, they tell me they are going to put me to work light jobs, but in the end I end up working the same thing… He told me that he was going to talk to my representative, but in the end, they didn't give me anything.

In summary, a combination of factors, including employers pressuring injured workers to continue working or RTW early, and workers not wanting to lose pay or disappoint their employers, combined to pressure many injured workers to continue working despite risks to their recovery. Some kept going until the pain became unbearable. Others were fired or decided to quit entirely if they could not manage their employer's demands. Almost universally, in one way or another, the RTW process failed injured workers interviewed for this study.

### Communication and Bureaucratic Challenges with WC Agencies

In Ontario, many participants reported a lack of communication from, and poor treatment by, WSIB case managers (e.g., apathy and disrespectful tone or language). For Andre, a worker from Jamaica, the lack of communication from his WSIB case manager meant he could not properly understand the letters he received in the mail:

Well, they could have been better in a way by explaining or communicating with me more. But in my terms, I will say, they don’t care… For one, there's no communication from them to me. And even though they are sending letters, sending letters upon letters, they are still not making any sense to me.

Indeed, many participants in both provinces reported challenges communicating with WC case managers due to language barriers or the use of inaccessible words or phrases. Santiago, a worker from Mexico, explained the challenges he experienced throughout the WC claims process:

I would like the WSIB to have or have a translator there in the hospital, to attend to us. If we are Hispanic, should we speak English? [The] WSIB sent me some papers, two white envelopes arrived and one brown envelope… but when [the boss] gave it to me it was already open, [which] was also what I didn't like… Look, it's all in English… I don’t know English….

In both provinces, participants were confused by the effects of being reassigned to a new WC case manager; many felt these changes caused delays in processing their claims. In some cases, participants reported no communication with any case manager. For example, Juan, a worker from Guatemala injured in Quebec, explained: “Well, I have never communicated with any (from the CNESST), because the ones who supposedly do everything are the bosses.”

Changing WSIB case managers caused Carol, a worker from Jamaica, significant delays in accessing her benefits:

When I tried to contact the case worker, he was on leave. Then when I call back, it was a different case worker, and everything have to be starting all over again… I remember one of them [was] like full of attitude. I think I maybe changed about six case workers. It was like every six months it's a different person.

In summary, language differences, challenges reaching and communicating with WC staff, frequent turnover of case managers, and paperwork that workers found overly complex or unintelligible were prominent issues that further challenged their ability to access WC benefits and programs, including RTW.

### Long-Term Impacts on Workers

Participants consistently reported ongoing pain and suffering after their job-related illness or injury in Canada and various long-term impacts related to inadequate WC and RTW support. Prolonged unemployment and poverty were prominent among our findings, due to pain and disability. For example, more than a decade after being hurt on a Quebec job in 2012, Luis, from Guatemala, explained:

The truth is, to this day my knee hurts, I have a pain in my knee because I never received anything from a hospital or therapy, or anything like that. I still have that pain in my knee, it hurts now, imagine, that was in 2012.

Likewise, Carol explained how pain from the injury she got working in Ontario agriculture curtailed her plans for the future and impacted all areas of her life:

My goal was to come to Canada to work for the period of time because I always wanted to be a nurse. Now everything has changed because I am not able to hold even a pen too long to write a letter or a note. If I’m on the computer, I’m not able to hold up my hand too long to type anything…. even to comb my hair it's a struggle. So, that really break me to know that I come, and I work so hard and when I get injured on Canadian soil that I will be treated unfairly.

Similarly, ongoing pain from a serious back injury caused by faulty farm equipment forced Kevin to return home to Jamaica in 2015. He still has chronic pain and cannot work or provide for his family:

I’m the breadwinner for the family. When I leave for Canada, it was like an opportunity. Then me go and me get hurt. Coming back home to them now like sometimes it's kind of frustrating to – knowing that I can’t do much anymore for them.

Given the severity of his injury, Kevin could not understand why he did not get WC or help to recover his health or income-earning ability. As a result, he and his family experienced food insecurity and could no longer afford to keep the children in school, which in turn caused him psychological distress:

What WSIB did to me is a wickedness, cause them make me hungry over there. My children over in Jamaica struggled, they didn’t even have food, my children could not go to school for a whole time, because of them people [at WSIB]. So, they traumatize my whole life. And I know those people are professional, and they operate as if they weren’t… they make my children go through trauma, for their whole life they will feel that still.

In sum, seriously injured or ill workers across all countries of origin, and in both provinces, emphasized common outcomes. Their job-related illnesses and/or injuries not only limited their physical capacity and/or led to chronic pain, they also resulted in loss of employment, loss of income, and the inability to support their families or fulfill life goals. In these situations, inadequate compensation, and lack of support to retrain and return to any form of work, has profound long-term impacts on workers’ financial, mental, and physical well-being, and their sense of purpose, as well as their families’ well-being and long-term prospects.

## Discussion

Although eligible for WC benefits and programs, the conditions under which migrant workers are employed through the streams of Canada's TFWP often render these unattainable. Like the challenges faced by vulnerable resident workers, migrant workers in Canada are subject to three added “layers of vulnerability” related to: migration factors (i.e., migration status and recruitment conditions); characteristics related to migrants and their country of origin (e.g., education and skills level, language skills); and receiving country conditions, including employment and sector characteristics, regulatory protections, access to collective representation, and issues of social exclusion/social isolation.^25^ We found that RTW policies in both provinces fail to address the unique and compounded challenges facing migrant agricultural workers due to their precarious employment conditions and their precarious immigration status as deportable noncitizens.

As noted, TFWP work permits in agriculture are employer-specific, meaning the worker can only work for the employer named on the permit (unless a transfer is arranged, which typically requires the employer's approval). The repatriation clause in the contract that lets employers terminate migrant workers without cause or process is particularly problematic; workers often feel the need to conceal and work through health or injury issues out of fear of being sent home. This possibility of forced or coerced return, combined with the system whereby employers “name” workers so that they can return in subsequent years, means that migrant agricultural workers rely on employer endorsement and approval for current and future jobs. This creates precarity and insecurity for workers, making most of them reluctant to report abuse, which includes hazardous working conditions.^68,69^ In this sense, fear of repatriation becomes a disciplinary tool used against migrant agricultural workers in particular, generating a docile, disposable, and low-wage workforce.^70^ In rare cases, workers who have been abused in some way, and decide to leave the workplace, can turn to community organizations to help apply for an open work permit for vulnerable workers.^71^ Those who do not know any community association and who give up their jobs, including several in our sample, often become part of the undocumented labor force, adding another layer of vulnerability.

When a migrant worker suffers a job-related illness or injury in Canada and cannot work, employers are typically concerned about the impact on production and WC premium costs, so they pressure injured workers to return to their job duties before they should. The fact that many participants reported that their employers agreed to the RTW recommendations set out by the provincial WC agency, but later failed to implement these obligations, speaks to the power imbalance between employers and migrant workers. The same fears of immediate repatriation and loss of future employment that maintain workers’ silence in the face of OHS hazards and abuse likewise discourage injured workers from reporting employers who violate their legal obligations in the RTW process.

Before hiring a migrant worker, Canadian employers must obtain a Labor Market Impact Assessment (LMIA) from the federal government, confirming that no Canadian worker is available to fill the position. Employers then are approved to hire a specific number of foreign migrant workers in a given year. If an injured migrant worker cannot do their usual job, employers often choose to terminate and repatriate them and try to get a replacement using the same LMIA approval. If the injured worker stayed in Canada, and their employer already had hired their maximum allotment of migrant workers, the employer might need to apply for a new LMIA to hire another worker and start the long bureaucratic hiring process all over again, including arranging housing.

For this reason, the TFWP repatriation clause incentivizes employers to repatriate injured migrant workers to maintain optimal business operations. There are additional financial incentives for employers to repatriate injured workers (e.g., to avoid costs associated with medical care and housing and potential legal liabilities). Furthermore, in agriculture, “light duties” are not always a feasible option; having a worker employed in modified tasks often means they are not able to meaningfully contribute to the required tasks, yet the employer still must pay them.

Once a worker is returned to their home country, the employer has no obligation to rehire them the following season, given their lack of job security as temporary contract employees. The Ontario and Quebec WC agencies’ RTW policies fail to account for the significant and multifaceted ways that “deportability”^70^ impedes injured migrant workers’ ability to recover their health and get back to work. Furthermore, WC agencies do not adequately make their correspondence accessible to migrant workers, who often do not speak English, have low levels of formal education, and/or experience challenges getting letters, particularly when mediated by employers or once repatriated. Likewise, the TFWP, designed at the federal level to fulfill employers’ needs, fails to consider how injured workers fall through the cracks of these two jurisdictional systems. Without adequate WC and RTW supports that recognize and account for these realities, most seriously injured migrant workers experience ongoing pain and suffering, unemployment, and poverty.

## Recommendations and Conclusion

As a key aspect of Canada's WC systems, the RTW process requires rethinking to align with the realities of the international migrant workforce and with international normative frameworks about human and labor rights. In particular, injured migrant workers require greater RTW supports on par with permanent resident/citizen workers, while also accounting for their unique realities. For example, they must be accessible and provided in the worker's preferred language, without interruption by repatriation.^60,72^ Workers who cannot return to their previous jobs in Canada should be given opportunities to retrain and reintegrate into the Canadian labor market. This will require cooperation between federal authorities who administer the TFWP and work permits and provincial ones who administer WC agencies and provide work permit approvals, as they do in Quebec. Instead, the current system wedges migrant workers between these two jurisdictions, with each government able to claim the responsibility or fault for the fate of injured, unemployed migrant workers belongs to the other.

To rectify these inequities, injured migrant workers should be able to stay in Canada to access all necessary health care and be supported to fully get back to work if possible. No injured worker should return home before health care is prearranged there. All correspondence with migrant workers should be provided by specially trained case managers who speak the worker's language (or who arrange high-quality interpretation) and can ensure the worker fully understands all correspondence. Injured or ill migrant workers should be referred to trained, independent legal advocates for assistance when needed; provincial governments should properly fund those advocates. Injured migrant workers who return home unable to work should be able to access their choice of health-care providers in their countries of origin, as part of the coverage by Canadian WC systems. As noted elsewhere in this special issue (Yachnin), WC agencies across Canada must provide all injured migrant workers with fair loss of earning benefits that recognize their local labor market realities, regardless of the TFWP stream in which they are employed.^b^ Further, migrant workers should be offered the chance to meaningfully retrain and reintegrate into the Canadian labor market if they wish, in a sector outside of agriculture if necessary.

Without these changes, this outdated system will continue to push migrant workers to work through injury and illness, through unhealthy and unsafe working conditions, further exacerbating their injuries and illnesses. When the harm is so severe they can no longer work or ignore the hazardous conditions, or when they are fired, they are forced to return to their countries of origin instead of being supported to retrain and recover. They then are typically unable to return to work in Canada (since they would no longer qualify as fit to work) or even in their home country, where labor market re-entry opportunities are limited, particularly for those with physical limitations. For some, the best option may be to remain in Canada and become an undocumented worker.

Injured migrant workers (and their families) bear the brunt of these structural barriers, quickly and quietly replaced with able-bodied workers, while they are relegated to suffering in pain and poverty, often for the rest of their lives. Too often, such workers are rendered disposable by a system that was never designed for them. It's a system that should have the disclaimer “no return,” since the promise of “return to work” clearly is unrealizable.

## Supplemental Material

sj-docx-1-new-10.1177_10482911251314149 - Supplemental material for The Point of No Return? Impediments to Return to Work for Injured Migrant Agricultural Workers in Two Canadian Provinces Supplemental material, sj-docx-1-new-10.1177_10482911251314149 for The Point of No Return? Impediments to Return to Work for Injured Migrant Agricultural Workers in Two Canadian Provinces by Stephanie Mayell, Janet McLaughlin, Jenna Hennebry, Guillermo Ventura Sanchez, Pankil Goswami and Jill Hanley in NEW SOLUTIONS: A Journal of Environmental and Occupational Health Policy

sj-docx-2-new-10.1177_10482911251314149 - Supplemental material for The Point of No Return? Impediments to Return to Work for Injured Migrant Agricultural Workers in Two Canadian Provinces Supplemental material, sj-docx-2-new-10.1177_10482911251314149 for The Point of No Return? Impediments to Return to Work for Injured Migrant Agricultural Workers in Two Canadian Provinces by Stephanie Mayell, Janet McLaughlin, Jenna Hennebry, Guillermo Ventura Sanchez, Pankil Goswami and Jill Hanley in NEW SOLUTIONS: A Journal of Environmental and Occupational Health Policy

sj-pdf-1-new-10.1177_10482911251314149 - Supplemental material for The Point of No Return? Impediments to Return to Work for Injured Migrant Agricultural Workers in Two Canadian Provinces Supplemental material, sj-pdf-1-new-10.1177_10482911251314149 for The Point of No Return? Impediments to Return to Work for Injured Migrant Agricultural Workers in Two Canadian Provinces by Stephanie Mayell, Janet McLaughlin, Jenna Hennebry, Guillermo Ventura Sanchez, Pankil Goswami and Jill Hanley in NEW SOLUTIONS: A Journal of Environmental and Occupational Health Policy

## Notes

a. *Policy and practice in return to work after work injury: Challenging circumstances and innovative solutions*. The project included a multidisciplinary team of academics, partners, and trainees from various universities to investigate how Canadian public policies can promote better practices to ensure a sustainable return to work for all injured workers.

b. In a recent hopeful step in the right direction, in September 2023, the Ontario Provincial Appeals Tribunal ordered the Ontario WSIB to compensate some injured migrant workers for their lost wages according to the realities of the job market in their countries of origin and their inability to return to Ontario (Mayell et al., 2024). WSIB's new strategy includes changes to how the board reviews suitable work available for injured migrant workers based on the labor market realities in their home countries. The board also committed to paying out millions of dollars to migrant workers who have been injured since 2007 while working under the Seasonal Agricultural Worker Program (SAWP). Although a notable step in the right direction, many migrant workers are excluded from this plan, including those injured before 2007 and workers employed under the other streams of Canada's Temporary Foreign Worker Program. To seriously address the injustices facing injured migrant workers, we recommend the WSIB expand their recent announcement to include all workers who have been injured in Ontario since the WSIB's practice of deeming began in the 1990s. Furthermore, these changes are not consistent across provinces in Canada, and it is imperative that provincial systems be aligned to ensure workers can fully and equally access their labor rights. Indeed, workers in Quebec must file a formal claim within six months of the employment injury, a deadline which is unknown to most workers, and many are repatriated once an injury is discovered. According to the *Loi sur les Normes du Travail* in section 92.9 “employers who hired a foreign temporary worker must inform without delay the arrival date and the contract time to the Commission, and if the departure date does not coincide with the end of the contract, the employer must inform the date and the reasons of the departure.” Additionally, Section 92.10 states that “if after investigation, the Commission has reasons to believe that a temporary foreign worker has been victim of violation of a right conferred by this law or regulation, the Commission could, even without a claim and if no other regulation intervenes, exercise any recourse in favor of the worker.” Those sections, as we have seen in our research, are not respected.

## References

[1] Government of Canada. The state of labour in agriculture and agri-food, https://agriculture.canada.ca/en/sector/data-reports/state-labour-agriculture-and-agri-food (2024, accessed 14 August 2024).

[2] Statistics Canada. Countries of citizenship for temporary foreign workers in the agricultural sector. https://www150.statcan.gc.ca/t1/tbl1/en/tv.action?pid=3210022101 (2024, accessed 14 August 2024).

[3] Organization for Economic Co-operation and Development (OECD). How does migration shape industry structure? In International Migration Outlook 2020. 2024. 10.1787/26a5b23b-en .

[4] Caxaj S Tran M Mayell S , et al. Migrant agricultural workers’ deaths in Ontario from January 2020 to June 2021: a qualitative descriptive study. Int J Equity Health 2022; 21: 98. 35842656 10.1186/s12939-022-01692-7 PMC9287708

[5] Hennebry JL . Not just a few bad apples: vulnerability, health and temporary migration in Canada. Canadian Issues/Themes Canadiens 2010; 1: 73–77.

[6] Mayell S . “It’s like we’re still in slavery”: stress as distress and discourse among Jamaican farm workers in Ontario, Canada. Soc Sci 2024; 13: 16. doi:10.3390/socsci13010016

[7] McLaughlin J . Trouble in our fields: health and human rights among Canada’s foreign migrant agricultural workers . PhD Dissertation, University of Toronto, Canada, 2009.

[8] McLaughlin J Hennebry J . Pathways to precarity: structural vulnerabilities and lived consequences in the everyday lives of migrant farmworkers in Canada. In: Producing and negotiating non-citizenship: precarious legal status in Canada. Waterloo: International Migration Research Centre, Wilfrid Laurier University, 2013, pp.174–194.

[9] Lu Y Hou F . Temporary foreign workers in the Canadian labour force: open versus employer-specific work permits. Report for Statistics Canada/Statistique Canada. 2019.

[10] Caxaj CS Weiler AM Martyniuk J . Housing conditions and health implications for migrant agricultural workers in Canada: a scoping review. CJNR 2024; 56(1): 16–28. 37844611 10.1177/08445621231203086 PMC10804689

[11] Canadian Agricultural Injury Reporting (CAIR). Agriculture-Related Fatalities in Canada: 1990–2020, https://www.casa-acsa.ca/wp-content/uploads/CAIR_Ag_Fatalities_1990-2020_en_V6.pdf (2023, accessed 14 August 2024).

[12] Moyce SC Schenker M . Migrant workers and their occupational health and safety. Annu rev Public Health 2018; 39(1): 351–365. 29400993 10.1146/annurev-publhealth-040617-013714

[13] Commission des normes, de l'équité, de la santé et de la sécurité du travail (CNESST). Agriculture. https://www.cnesst.gouv.qc.ca/fr/prevention-securite/informations-prevention/prevention-par-secteur-dactivite/agriculture#:∼:text=Les%20métiers%20de%20l'agriculture,le%20bien%2Dêtre%20des%20travailleurs, (2024, accessed 29 July 2024).

[14] Pysklywec M McLaughlin J Tew M , et al. Doctors within borders: meeting the health care needs of migrant farm workers in Canada. CMAJ 2011; 183(9): 1039–1042. 21502349 10.1503/cmaj.091404 PMC3114895

[15] Cole DC McLaughlin JE Hennebry JL , et al. Precarious patients: health professionals’ perspectives on providing care to Mexican and Jamaican migrants in Canada’s Seasonal Agricultural Worker Program. RRH 2019; 19(4): 1–10. 10.22605/RRH5313 31785605

[16] Curl CL Spivak M Phinney R , et al. Synthetic pesticides and health in vulnerable populations: agricultural workers. Curr Environ Health Rep 2020; 7(1): 13–29. 31960353 10.1007/s40572-020-00266-5 PMC7035203

[17] Kachuri L Harris MA MacLeod JS , et al. Cancer risks in a population-based study of 70,570 agricultural workers: results from the Canadian census health and environment cohort (CanCHEC). BMC Cancer 2017; 17(1): 1–15. 28525996 10.1186/s12885-017-3346-x PMC5437486

[18] Adam-Poupart A Labreche F Busque MA , et al. Association between outdoor ozone and compensated acute respiratory diseases among workers in Quebec (Canada). Ind Health 2015; 53(1): 171–175. 25736778 10.2486/indhealth.2014-0136 PMC4380604

[19] Colindres C Cohen A Caxaj CS . Migrant agricultural workers’ health, safety and access to protections: a descriptive survey identifying structural gaps and vulnerabilities in the interior of British Columbia, Canada. Int J Environ Res Public Health 2021; 18(7): 3696. 33916232 10.3390/ijerph18073696 PMC8037260

[20] Dabrowska-Miciula E de Lima P . Challenging a “cycle of neglect”: health and safety among transnational agricultural workers in Canada and the UK. In: Díaz Bretones F and Santos A (eds) Health, safety and well-being of migrant workers: new hazards, new workers. Cham: Springer International Publishing, 2020, pp.137–156.

[21] McLaughlin J Hennebry J Haines T . Paper versus practice: occupational health and safety protections and realities for temporary foreign agricultural workers in Ontario. PISTES 2014; 16(2): 1–20.

[22] Commission des normes, de l'équité, de la santé et de la sécurité du travail (CNESST). Agriculture, https://www.cnesst.gouv.qc.ca/fr/prevention-securite/informations-prevention/prevention-par-secteur-dactivite/agriculture, (2021, accessed 30 July 2024).

[23] Province of Ontario. Agriculture, growing, breeding, keeping and fishing, https://www.ontario.ca/document/industries-and-jobs-exemptions-or-special-rules/agriculture-growing-breeding-keeping-and-fishing#section-0, (2024, accessed 14 August 2024).

[24] McLaughlin J Hennebry J Cole D , et al. The migrant farmworker health journey: stages and strategies. In: IMRC policy points. Waterloo: International Migration Research Center, Wilfrid Laurier University, 2014. https://scholars.wlu.ca/cgi/viewcontent.cgi?article=1001&context=imrc .

[25] Sargeant M Tucker E . Layers of vulnerability in occupational safety and health for migrant workers: case studies from Canada and the UK. PPHS 2009; 7(2): 51–73.

[26] Vosko LF Tucker E Casey R . Enforcing employment standards for temporary migrant agricultural workers in Ontario, Canada: exposing underexplored layers of vulnerability. IJCCLIR 2019; 35(2): 227–254.

[27] Dehaibi L . Idéal champêtre ou enfer ouvrier? Démystifier la terre pour renforcer les droits des travailleurs agricoles. Labour 2022; 90(1): 111–147.

[28] Hanley J Paul L Ravinthiran J , et al. Protecting the rights of migrant farmworkers in Quebec: to what extent can unionization overcome the effects of precarious immigration status? JRCD 2020; 15(2): 122–146.

[29] Martinez S . Give thanks for migrant farm workers: protect them under Labour Relations Act. Law360 Canada. 2022 Oct 1. https://www.law360.ca/articles/40255 .

[30] Province of Ontario. Occupational Health and Safety Act (OHSA), https://www.ontario.ca/page/occupational-health-and-safety-act-ohsa, (2024, accessed 14 August 2024).

[31] Province of Quebec. Loi sur la santé et la sécurité du travail, https://www.legisquebec.gouv.qc.ca/fr/document/lc/s-2.1 (2024, accessed 14 August 2024).

[32] Barnes N . Is health a labour, citizenship or human right? Mexican seasonal agricultural workers in Leamington, Canada. Glob Public Health 2013; 8(6): 654–669. 23672480 10.1080/17441692.2013.791335

[33] Cedillo L Lippel K Nakache D . Factors influencing the health and safety of temporary foreign workers in skilled and low-skilled occupations in Canada. New Solut 2019; 29(3): 422–458. 31390284 10.1177/1048291119867757

[34] Côté D White B Dubé J , et al. Are healthcare systems failing immigrants? Transnational migration and social exclusion in the workers’ compensation process in Québec. IMR 2023. DOI: 10.1177/01979183231180192.

[35] Hanley J Gravel S Lippel K , et al. Pathways to healthcare for migrant workers: how can health entitlement influence occupational health trajectories? PISTES 2014; 16(2): 1–21.

[36] Wells D McLaughlin J Lyn A , et al. Sustaining precarious transnational families: the significance of remittances from Canada’s Seasonal Agricultural Workers Program. Just Labour 2014; 22(1): 144–167.

[37] Province of Ontario. Apply for OHIP and get a health card, https://www.ontario.ca/page/apply-ohip-and-get-health-card#section-2 (2017, accessed 5 August 2024).

[38] Province of Quebec. Know the eligibility conditions for health insurance, https://www.ramq.gouv.qc.ca/en/citizens/health-insurance/know-eligibility-conditions (2020, accessed 5 August 2024).

[39] Hennebry J McLaughlin J . The exception that proves the rule: structural vulnerability, health risks and consequences for temporary migrant farmworkers in Canada. In: Leonard PT Straehle C (eds) Legislated inequality: temporary labour migration in Canada. Montreal: McGill-Queen’s University Press, 2012, pp.117–138.

[40] McLaughlin J Tew M Huesca E . Compounded vulnerabilities and creative strategies: occupational health of temporary foreign agricultural workers. In: Premji S (ed) Sick and tired: health and safety inequalities. Halifax: Fernwood, 2018, pp.129–142.

[41] Province of Quebec. Work in the agricultural sector in Québec, https://www.ramq.gouv.qc.ca/en/citizens/health-insurance/register/r3-q1r2 (2020, accessed 3 August 2024).

[42] McLaughlin J Tew M . Migrant farm worker health care: unique strategies for a unique population. In: Arya AN Piggott T (eds) Under-served: health determinants of Indigenous, inner-city, and migrant populations in Canada. Toronto: Canadian Scholars, 2018, pp.253–264.

[43] Beatson J Hanley J . The intersection of exploitation and coercion in cases of Canadian labour trafficking. JLS 2017; 26(1): 137–158.

[44] Hennebry J McLaughlin J Preibisch K . Out of the loop: (in) access to health care for migrant workers in Canada. J Int Migr Integr 2016; 17(1): 521–538.

[45] Hennebry JL Preibisch K . A model for managed migration? Re-examining best practices in Canada’s seasonal agricultural worker program. IM 2012; 50(s1): e19–e40.

[46] Seto Nielsen L Goldstein Z Leung D , et al. A scoping review of undocumented immigrants and palliative care: implications for the Canadian context. J Immigr Minor Health 2019; 21(1): 1394–1405. 30982203 10.1007/s10903-019-00882-w

[47] Orkin AM Lay M McLaughlin J , et al. Medical repatriation of migrant farm workers in Ontario: a descriptive analysis. CMAJ Open 2014; 2(3): E192–E198. 10.9778/cmajo.20140014 PMC4183168 25295239

[48] Mayell S . We are like broken vessels; we get thrown out”: the triple violence of injury among Jamaican migrant farm workers in Canada. Dialec Anthropol 2024; 22(1): 1–24.

[49] Mayell S McLaughlin J . Migrating to work at what cost? The cumulative health consequences of contemporary labour migration. In: Thomas F (ed) Handbook of migration and health. Cheltenham: Edward Elgar Publishing, 2016, pp.230–252.

[50] Narushima M McLaughlin J Barrett-Greene J . Needs, risks, and context in sexual health among temporary foreign migrant farmworkers in Canada: a pilot study with Mexican and Caribbean workers. J Immigr Minor Health 2016; 18(1): 374–381. 25784142 10.1007/s10903-015-0189-x

[51] Salami B Meharali S Salami A . The health of temporary foreign workers in Canada: a scoping review. CJPH 2015; 106(1): e546–e554. 10.17269/CJPH.106.5182 PMC6972066 26986918

[52] Hennebry J McLaughlin J . Responding to temporary migration in Ontario’s agricultural workplaces. Final Research Report Submitted to the WSIB Research Advisory Council, 2012.

[53] Province of Ontario. Workplace Safety and Insurance Act, https://www.ontario.ca/laws/statute/97w16 (1997, accessed 29 July 2024).

[54] Province of Quebec. Act respecting industrial accidents and occupational diseases, https://www.legisquebec.gouv.qc.ca/en/document/cs/a-3.001 (1999, accessed 3 December 2024).

[55] Canadian Centre for Occupational Health and Safety (CCOHS). Return to work, https://www.ccohs.ca/oshanswers/psychosocial/rtw/rtw_program.html (2024, accessed 14 August 2024).

[56] Commission des normes, de l'équité, de la santé et de la sécurité du travail (CNESST). Return to work. https://www.cnesst.gouv.qc.ca/en/prevention-and-safety/healthy-workplace/return-work (2024, accessed 14 August 2024).

[57] Workplace Safety and Insurance Board (WSIB). RTW overview and key concepts. https://www.wsib.ca/en/operational-policy-manual/rtw-overview-and-key-concepts (2020, accessed 12 August 2024).

[58] Endicott M Mantis S . Who killed Sir William?: a community-university research alliance seeks justice for injured workers. Altona: Friesen Press, 2024.

[59] Premji S . Immigrant men’s women’s occupational health: questioning the myths. In: Premji S (ed) Sick and tired: health and safety inequalities. Halifax: Fernwood, 2018, pp.105–116.

[60] Premji S . Barriers to return-to-work for linguistic minorities in Ontario: an analysis of narratives from appeal decisions. J Occup Rehabil 2015; 25(1): 357–367. 25240395 10.1007/s10926-014-9544-3

[61] Gravel S Vissandjée B Lippel K , et al. Ethics and the compensation of immigrant workers for work-related injuries and illnesses. J Immigr Minor Health 2010; 12(1): 707–714. 19308731 10.1007/s10903-008-9208-5

[62] Premji S Begum M Medley A , et al. Return-to-work in a language barrier context: comparing Quebec’s and Ontario’s workers’ compensation policies and practices. PISTES 2021; 23(1): 1–22.

[63] Ricard-Guay A Hanley J Montgomery C , et al. Mère et sans-papier au Québec. In: Kanouté F Lafortune G (eds) L’integration des families d’origine immigrante. Les enjeux sociosanitaires et scolaires. Montreal: Les Presses de l’Université de Montréal, 2018, pp.49–64.

[64] Côté D Dubé J Gravel S , et al. Cumulative stigma among injured immigrant workers: a qualitative exploratory study in Montreal (Quebec, Canada). Disabil Rehabil 2020; 42(8): 1153–1166. 30686038 10.1080/09638288.2018.1517281

[65] Senthanar S Koehoorn M Tamburic L , et al. Differences in modified-return-to-work by immigration characteristics among a cohort of workers in British Columbia, Canada. J Occup Rehabil 2023; 33(1): 341–351. 36308629 10.1007/s10926-022-10077-0

[66] Workplace Safety and Insurance Board (WSIB). Report an injury or illness, https://www.wsib.ca/en/businesses/claims/report-injury-or-illness (2024, accessed 23 July 2024).

[67] Éducaloi. Workplace accidents: steps to take, remedies and compensation, https://educaloi.qc.ca/en/capsules/workplace-accidents-steps-to-take-remedies-and-compensation/ (2024, accessed 28 July 2024).

[68] Basok T Bélanger D . Migration management, disciplinary power, and performances of subjectivity: agricultural migrant workers’ in Ontario. CJS 2016; 41(2): 139–164.

[69] Preibisch K Hennebry J . Temporary migration, chronic effects: the health of international migrant workers in Canada. CMAJ 2011; 183(9): 1033–1038. 21502343 10.1503/cmaj.090736 PMC3114894

[70] Basok T Bélanger D Rivas E . Reproducing deportability: migrant agricultural workers in south-western Ontario. JEMS 2014; 40(9): 1394–1413.

[71] Government of Canada. Open work permit for vulnerable foreign workers who are victims of abuse, https://www.canada.ca/en/immigration-refugees-citizenship/services/work-canada/permit/temporary/vulnerable-workers.html (2022, accessed 28 July 2024).

[72] Coutu MF Durand MJ Coté D , et al. Ethnocultural minority workers and sustainable return to work following work disability: a qualitative interpretive description study. J Occup Rehabil 2022; 32(1): 773–789. 35616770 10.1007/s10926-022-10044-9

