# Supplementary material for: The Point of No Return? Impediments to Return to Work for Injured Migrant Agricultural Workers in Two Canadian Provinces
Source: New Solut. 2025 Feb 21;35(1):22–32. doi: 10.1177/10482911251314149 (PMC11954361; doi:10.1177/10482911251314149)
Supplement: sj-docx-2-new-10.1177_10482911251314149 - Supplemental material for The Point of No Return? Impediments to Return to Work for Injured Migrant Agricultural Workers in Two Canadian Provinces [file sj-docx-2-new-10.1177_10482911251314149.docx]

**INTERVIEW GUIDE FOR INJURED MIGRANT WORKERS**

Preamble following informed verbal consent process to begin the interview. As you know, we are interested in your experiences of return to work after a work injury/illness. We want to better understand your experiences with workers’ compensation, and the process of returning to work after an injury or illness in Canada. If it’s okay with you, we are going to start the interview now, and I’m going to record what we say so that we can remember it later. If there is any question or term you don’t understand, please let me know and I can explain it differently, and you can skip any questions or stop answering questions at any time. Do you have any questions before we begin? May I press record now?

INTERVIEW GUIDE – POSSIBLE QUESTIONS

Reminder to interviewer: ***These interviews will be semi-structured and open-ended and may include variations on the questions below.*** Interviews should follow a natural flow and q***uestions will be adapted to avoid repetition*** of what a worker has already said, ***and to match the worker’s unique circumstances*** (for example, if a worker had a back injury, you can use the word “back injury” instead of “illness/injury”) Instructions to interviewer are in italics throughout. Please skip any questions that are not relevant or which ask about information the worker has already discussed to try to keep the interview as concise as possible.

1. DEMOGRAPHICS AND BACKGROUND INFORMATION:
2. Under what program were you working in Canada when you were injured/ill (e.g. SAWP/AG Stream)? How long had you been working in Canada? How long had you been working in Quebec?
3. What country do you come from? What is your age? Gender? Which languages do you speak?
4. In what town/city/region were you employed?
5. Please tell me about your job. What work were you doing when you were injured or ill? What was your weekly salary at the time of your injury/illness? How long was your contract supposed to last? What were your duties? What did you know about your labour rights (good and bad) before you were injured? Who did you learn that from?
6. INJURY/ILLNESS INFORMATION:
7. Was this your first workplace injury/illness? If not, what were your previous issues? Did you report/claim them?
8. In what month/year did you experience your most recent injury or illness? What is the nature of this illness/injury? Can you tell us more about what happened, the context, and how your symptoms developed? How long were you off work?
9. Did you seek medical care in Canada, if so, what kind, and when? Were you satisfied with the care you received? Did you have access to a paid interpreter when accessing health care? Did you face any challenges in accessing this care? Did you have provincial (RAMQ) or private health insurance? Did you have to pay for any of this treatment? If so, how much?
10. (If applicable for workers in home countries) Did you seek medical care in your home country (insert country name)? Did you have to pay for any of this treatment? If so, how much? Were you reimbursed, and if so, by whom? Were you satisfied with the care you received? Did you face any challenges in accessing this care?
11. How and when did you hear about CNESST? Who told you? (Was it before or after the injury/illness)?
12. Had you heard anything about the treatment of injured workers that impacted your decision whether or not to file for CNESST?

11) Did you file for workers’ compensation? If not, why not? (Skip section C).

1. QUESTIONS FOR WORKERS WHO FILED FOR CNESST

12) Did you consider not filing a claim? If so, why?

13) At what point after the onset of symptoms or after the injury was a claim filed with CNESST?

1. Did you file your own claim?
2. Did someone assist you with the CNESST claim process (e.g., application, correspondence, etc.) If so, who? (e.g., liaison/consulate, legal clinic, employer, other)
3. Did your employer also file a claim? Did you see the claim he or she filed? If so, did you agree with their assessment of what happened?
4. Did your physician/heath care provider file a claim? Did you see the claim he or she filed? If so, did you agree with their assessment of what happened?
5. Did you experience any problems during the claim process (probes: applying, difficulty receiving or understanding the correspondence, etc.)? If so, explain?
6. Did CNESST send you to a Bureau d’évaluation médicale (BEM) to evaluate your injury (or illness)? If so, were you happy with the treatment? Prognosis? Why or why not? What could have been improved? What was the ruling?
7. Have you heard from your CNESST case manager directly regarding your claim? If so, how were you contacted? Were you given the opportunity to tell your case manager directly about the details of your injury or illness?
8. Where was your correspondence delivered to? Did anyone else open the correspondence first? Did you feel your privacy/confidentiality was respected?
9. Did you receive loss of earnings compensation (i.e. money to replace your lost income for the days you weren’t working?) (If no, move to next question). How many days were you compensated for? Do you feel this was enough time off to recover? Did you receive enough money to support your needs during this time? Did you feel the money was sufficient to replace your income?
10. Did you receive benefits for non-economic loss for your injury (cater to the worker’s injury, such as money given to you to compensate for the loss of a limb) (to determine permanent impairment)? If so, how much did you receive? Did you understand and agree with the settlement you received? Why or why not?
11. Did you appeal your claim? If so, tell us the details. If not, why not?
12. Overall, do you feel you have been treated fairly by the CNESST? Why or why not?
13. When trying to access your settlement money from CNESST, did you experience any of the following: (read and have them indicate which apply)
    1. Any trouble receiving or understanding the communication or receiving compensation from CNESST while in Canada or after you returned home?
    2. How long did you wait to receive your compensation from the bank? Did this affect your ability to access health care or take care of your other needs?
    3. Any difficulty in providing any medical evidence or follow-up from outside the country?
    4. Did your situation in the SAWP or TFWP influence your claim or the process of filing the claim? (e.g. sent home before forms were filled out)?
    5. Other?
14. Did the COVID-19 pandemic affect your CNESST benefits or your return-to-work experience? If so in what way?
15. **WORKERS WITH COVID-19 (These questions to be asked only of workers who indicated their work-related illness was COVID-19):**
16. (If not already addressed sufficiently earlier): With respect to your COVID illness, can you tell us more about what happened, the context, and how your symptoms developed? (Probes: Did other workers get sick first? Do you know where you contracted it?)
17. At what point were you offered COVID testing, where and by whom? How was this experience?
18. Were you placed into quarantine? If so, where and for how long? How was that experience? Were you offered medical assistance during quarantine? If so, what did that look like? Were you needs, like food, taken care of? Were you able to talk to your family?
19. Had you been offered a vaccine? If so, can you tell us more about this experience? Which vaccine? Where? Who offered it? One dose or two? Did you take it? Why or why not?
20. Did you feel like there were sufficient safety precautions at your farm or living quarters to prevent COVID-19 transmission? Why or why not?
21. Did you hear about CNESST in relation to COVID-19 (that you could file for workers’ compensation for COVID) before or after you got COVID? How did you hear about it?
22. What symptoms did you experience in relation to COVID-19 and how long did they last?
23. How did you feel emotionally while having COVID-19? (e.g. sad, scared, angry, anxious, optimistic)
24. Is there anything else you would like to share with us about your return to work experience during COVID-19?
25. **RETURN TO WORK (RTW) QUESTIONS FOR ALL WORKERS:**
26. Tell me about your experience returning to work with your employer where you were injured. Was a formal return to work process arranged before your treatment was over? How did your return to work progress?
27. Did your employer arrange return to work for you once your doctor told you that you could go back to work? If not, why not? If so, please tell me how return to work was set up for you?
28. How did you manage your return to employment after you were injured? What were some tough parts and good parts?
29. What was your experience of trying to find a job after your injury? Did you return to the same employer? If no, why? Did someone help you find other employment? If so, who?
30. Did you have a medical person to go to if you had problems in returning to work (RTW)?
31. Was a labour union or any other organization involved in the return to work process? Was that helpful?
32. Were you asked to do modified work? Did you feel it was safe and suitable? Did you do the modified work? If so, how was it? If not, why not? What happened?

***Probes***:

a. How did your employer treat you?

b. How did your colleagues treat you?

c. Conflict with employer?

d. Pressure to return to work before you were ready?

e. Experience of racism or discrimination (due to injury or claim) from employer or work colleagues?

f. Were white/Canadian staff treated differently? If yes, how?

1. What other problems at your work location impacted your RTW (e.g. housing, employment conditions, modified work duties? (If applicable)
2. Were you repatriated after your injury/illness? If so, how quickly? Were you invited back to work the following season?
3. How was your living situation following your injury? Did you have a safe and comfortable place to recover? Were their limitations within your living situation? Did you have someone to care for you? What would have helped you to recover? ***Probes -*** Family? Quiet space?
4. Did you have to depend on employer for health care access?
5. Do you feel that your condition now is fully resolved? (In other words, are you 100% back to normal?) If not, what symptoms persist? How common and severe are they?
6. Are you still receiving medical treatment for your injury/illness? Why or why not? If not, do you think you should be?
7. How has this injury/illness affected your life circumstances? (e.g. your ability to work and undertake regular daily activities, your sleep, your family relationships, your financial stability, your mental/emotional wellbeing)
8. In addition to income, what else did you lose after the accident (e.g. housing, transportation into town, access to free food)?
9. Has this illness or injury prevented you from participating in the TFWP/SAWP or impacted your ability to come to Canada/stay in Canada?
10. We are also interested in how you managed return to work in relation to any family responsibilities you might have. Can you tell us about any aspects of work-life balance that were affected by your return to work arrangements? Did your family participate in helping you return to work?
11. In your ideal circumstance, what would the return to work process look like? What supports would you receive?
12. **RETURN TO WORK QUESTIONS ONLY FOR WORKERS WHO HAD A CNESST CLAIM:**
13. Did the CNESST play a part in your return to work process? If not, why not? If so, what were some good parts? Tough parts?

a. Did you interact with a RTW specialist?

***Probes:*** If yes, what happened? what did they say? How did they treat you?

1. Was there an appeal to the Tribunal administratif du travail (TAT) concerning the employment proposed to you or concerning the occupational rehabilitation process or the actual experience of returning to work? If so, please describe your experience in appeal.
2. Could you speak freely to CNESST about any problems you experienced with return to work? Why or why not? Were your concerns considered by CNESST in your return to work (arrangements)?
3. In an ideal world, how would you improve the system of workplace compensation? Why are such changes needed?

**FINAL STATEMENT FOR ALL WORKERS**

Thank you so much for your time. If there is anything else you’d like to add, please let me know. Otherwise, the interview is complete
