# Supplementary material for: The Point of No Return? Impediments to Return to Work for Injured Migrant Agricultural Workers in Two Canadian Provinces
Source: New Solut. 2025 Feb 21;35(1):22–32. doi: 10.1177/10482911251314149 (PMC11954361; doi:10.1177/10482911251314149)
Supplement: sj-pdf-1-new-10.1177_10482911251314149 - Supplemental material for The Point of No Return? Impediments to Return to Work for Injured Migrant Agricultural Workers in Two Canadian Provinces [file sj-pdf-1-new-10.1177_10482911251314149.pdf]

Return-to-Work Challenges Among International Migrant Agricultural Workers in Two Canadian Provinces

New Solutions Webinar

Sustainable Return to Work in Vulnerable Situation: Policy, Practice and the Canadian Context

June 9, 2025

Stephanie Mayell, MA
PhD Candidate, University of Toronto

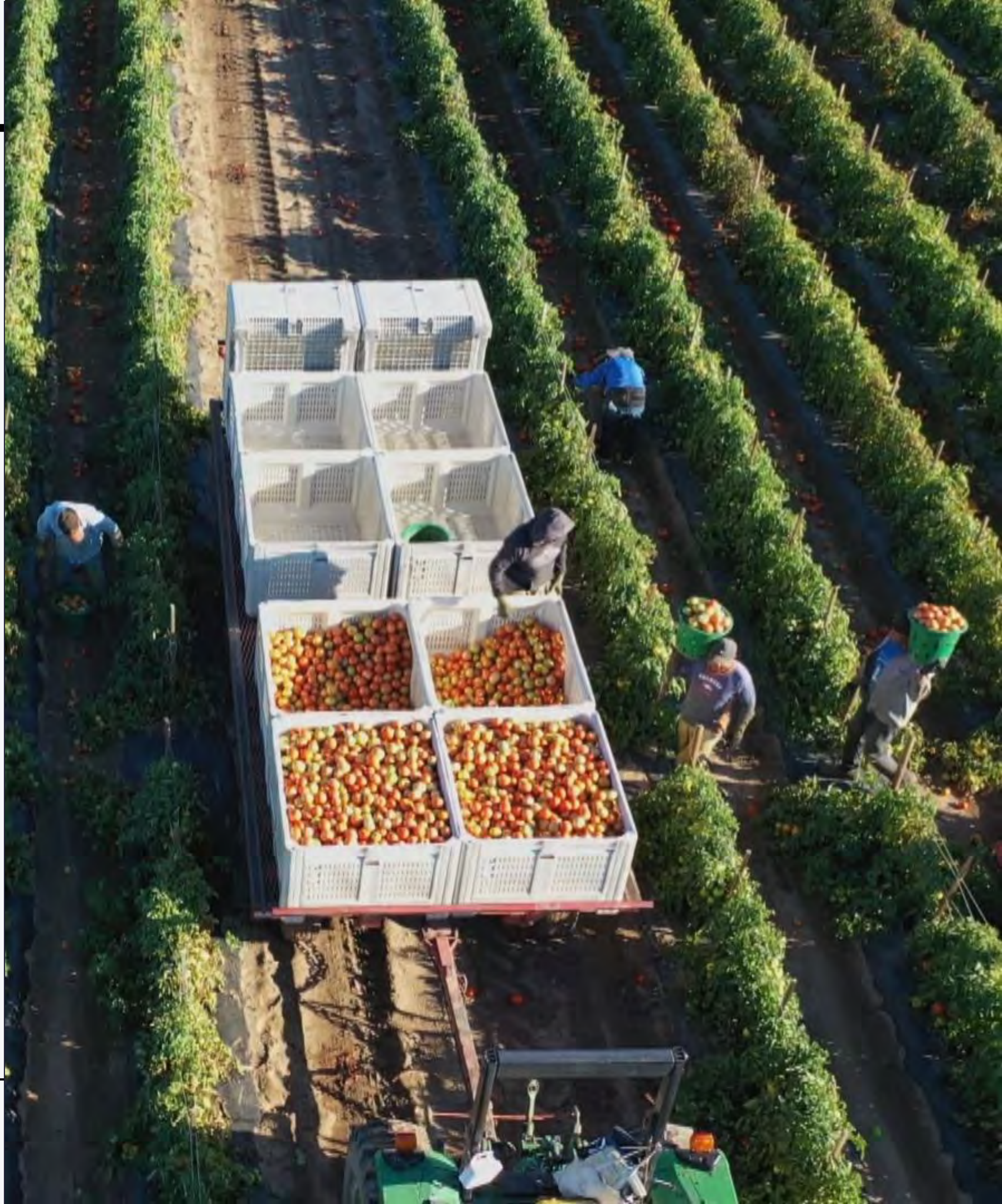

MIGRANT AGRICULTURAL WORKERS (MAWs) IN CANADA

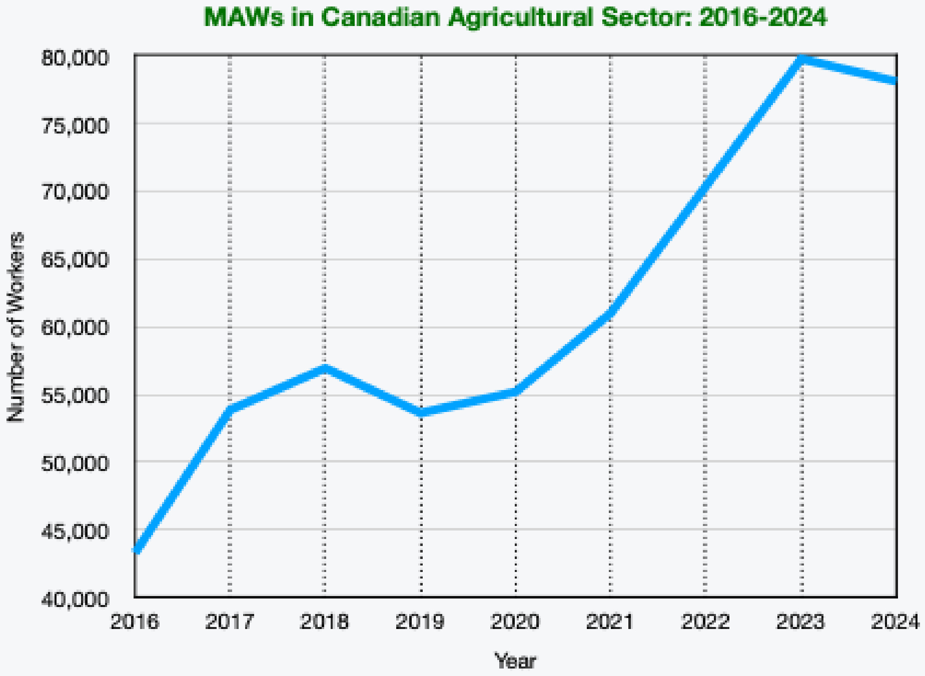

Source: https://www150.statcan.gc.ca/t1/tbl1/en/tv.action?pid=3210022101

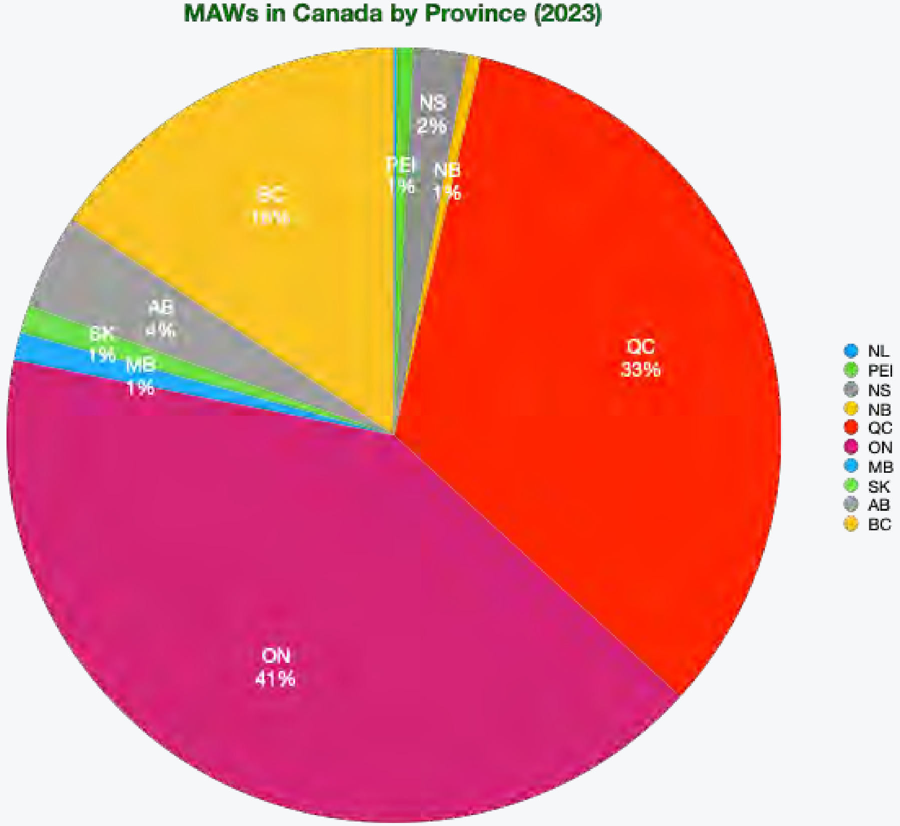

Source: https://agriculture.canada.ca/en/sector/data-reports/state-labour-agriculture-and-agri-food

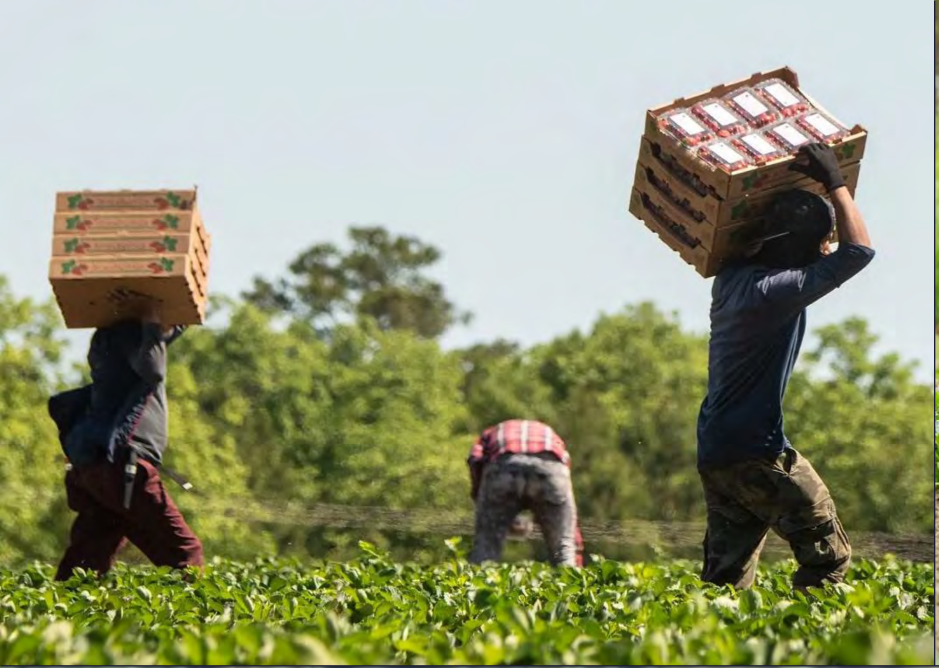
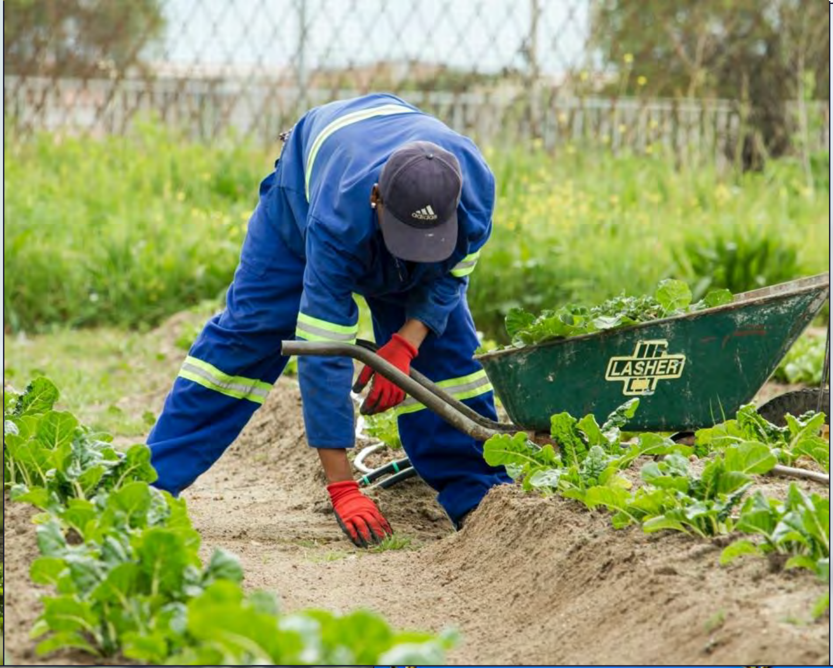
LASHER
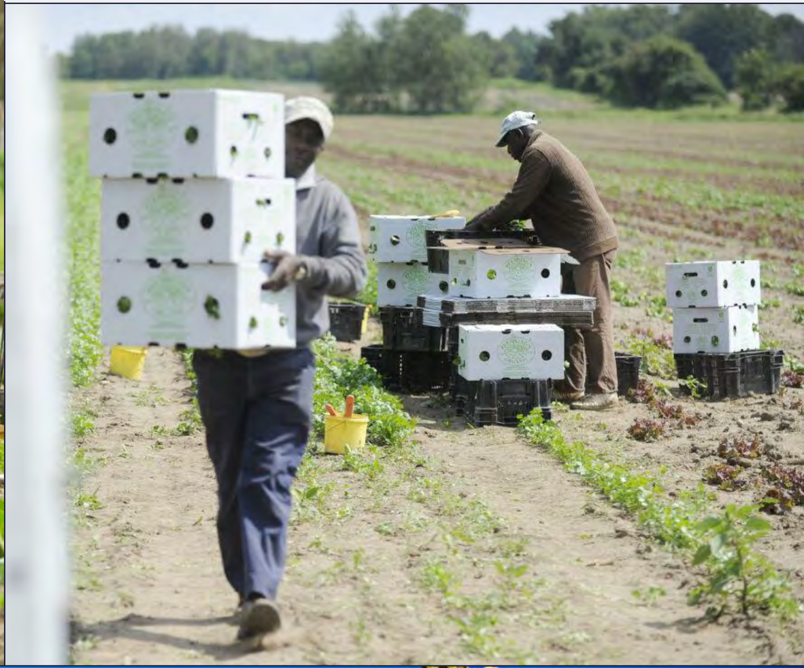
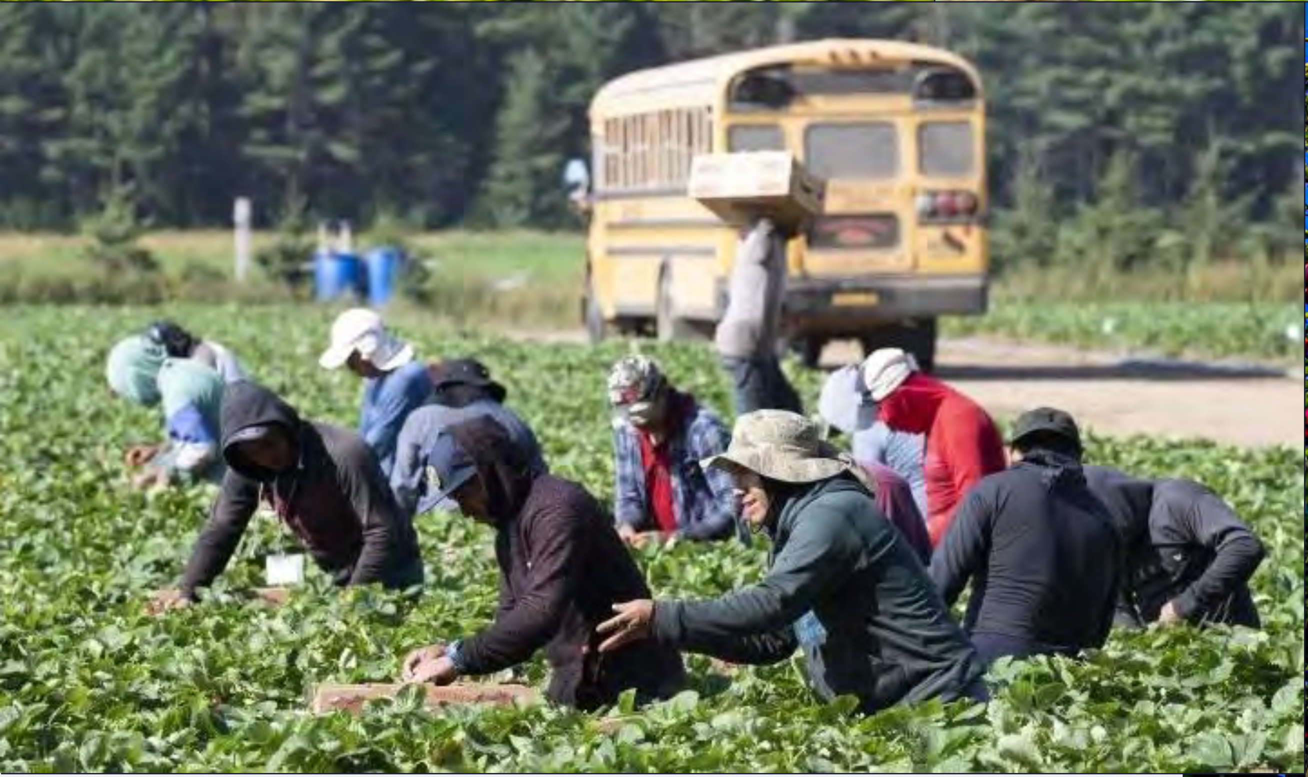
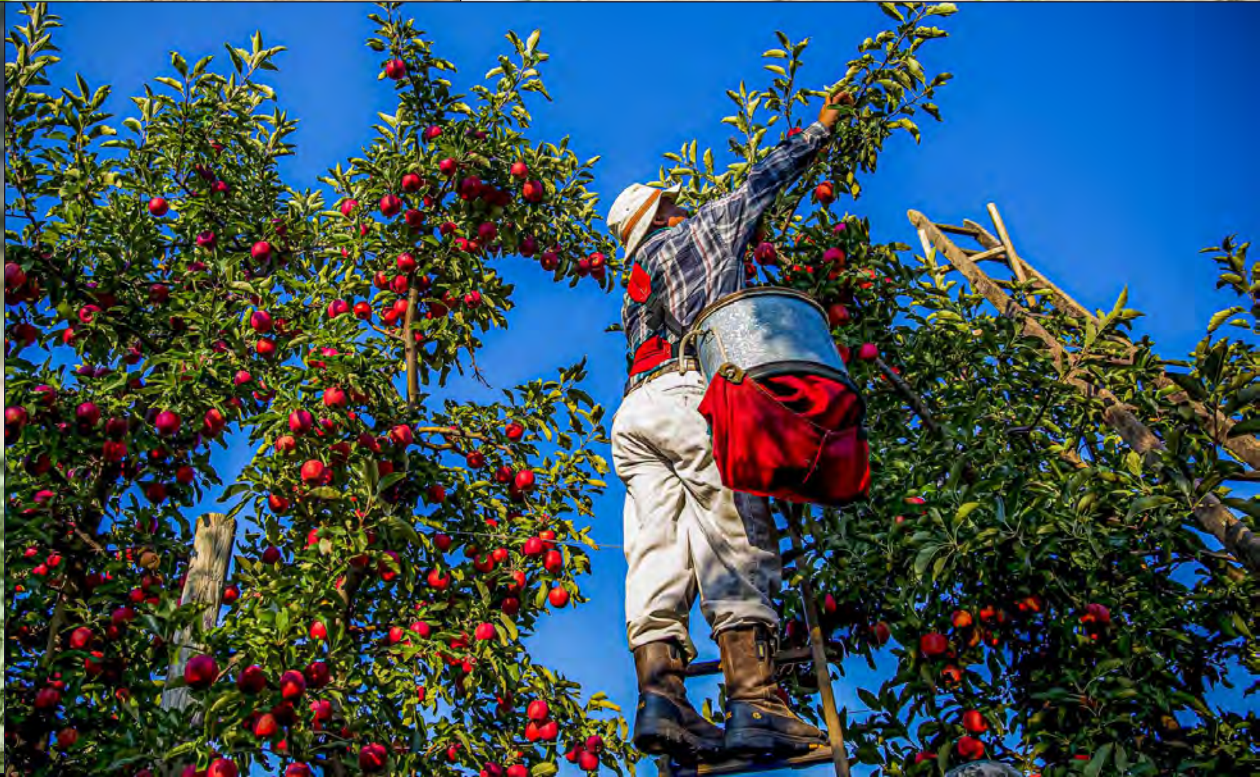

BARRIERS TO ACCESSING HEALTH CARE & WORKERS' COMPENSATION (WC) BENEFITS

- MAWs entitled to provincial health insurance, but face many barriers
- Compounded factors impact WC (and RTW) rates among MAWs
- MAWs often repatriated before receiving adequate health care or compensation

RESEARCH GOALS

The goals of our research were twofold:

1. To identify the barriers and facilitators that affect return-to-work (RTW) outcomes for sick and injured migrant workers across various sectors and streams of the Temporary Foreign Worker Program (TFWP).
2. To develop policy recommendations that promote optimal RTW outcomes for migrant workers who are injured or become ill in Ontario and Quebec.

METHODOLOGY

Our methodology included two main components:

1. An analysis of WC policies in Ontario and Quebec.
2. Semi-structured interviews with MAWs (n=44) in these provinces.

Interviews focused on several key themes:

- Pre-injury work conditions
- Nature and circumstances of workplace injury or illness
- Aftermath (e.g. employers' reactions, health consequences, access to health care)
- Interactions with WC agency
- The RTW process

FINDINGS: BARRIERS TO RTW AMONG MAWs

- Injured or ill MAWs in Ontario and Quebec face significant challenges accessing RTW services, affecting both health and employment.
- **Our findings are organised into three main themes:**

1. Pressure to Return to Work Prematurely
2. Communication & Bureaucratic Barriers
3. Long-Term Impacts of Failed RTW

PRESSURE TO RETURN TO WORK PREMATURELY

"[It] was documented in the file that I am doing light duty, which wasn't so. I went back out in the field…that day I actually was partially paralyzed by midday, I couldn't walk, couldn't nothing. I had to be lifted to the boss, and they did not take me to the doctor."

- Garfield, worker from Jamaica

COMMUNICATION & BUREAUCRATIC BARRIERS

"I would like the WSIB to have a translator… If we are Hispanic, should we speak English? [The] WSIB sent me some papers, two white envelopes arrived and one brown envelope… but when [the boss] gave it to me it was already open, [which] was also what I didn't like… Look, it's all in English… I don't know English…."

- Santiago, worker from Mexico

LONG-TERM IMPACTS OF FAILED RTW

"What WSIB did to me is a wickedness, cause them make me hungry over there. My children… they didn't even have food, my children could not go to school for a whole time, because of them people [at WSIB]. So, they traumatize my whole life. And I know those people are professional, and they operate as if they weren't… they make my children go through trauma, for their whole life they will feel that still."

- Kevin, worker from Jamaica

DISCUSSION

- Migrant workers face unique RTW challenges due to precarious employment, immigration status, and limited regulatory protections.
- Employer-specific permits and repatriation clause discourage injury reporting and create a climate of fear.
- WC and RTW policies fail to address the realities of "deportability" and communication barriers.
- Most injured MAWs experience ongoing hardship, unemployment, and poverty due to inadequate support and protections.

POLICY RECOMMENDATIONS

- Reform RTW process for equal support to MAW and alignment with human/labour rights
- Ensure RTW supports, retraining, and health care in preferred language, uninterrupted by repatriation
- Improve federal-provincial coordination of retraining and reintegration of injured MAWs into Canadian labour market
- Guarantee fair loss-of-earnings benefits and access to legal advocates in Canada and after return home

SSHRC CRSH
Social Sciences and Humanities Research Council
Conseil de recherches en sciences humaines

THANK YOU!

Stephanie Mayell, MA
PhD Candidate
Department of Anthropology,
University of Toronto
steph.mayell@gmail.com

Janet McLaughlin, PhD
Associate Professor
Department of Health Studies,
Wilfrid Laurier University
jmclaughlin@wlu.ca

Jenna Hennebry, PhD
Professor
International Migration Research Centre,
Wilfrid Laurier University
jhennebry@wlu.ca

Guillermo Ventura Sanchez
PhD Candidate
Dept. of Sociology and Anthropology,
Concordia University
g_ven@live.concordia.ca

Pankil Goswami
PhD Candidate
School of Social Work,
McGill University
pankil.goswami@mail.mcgill.ca

Jill Hanley, PhD
Associate Professor
School of Social Work,
McGill University
jill.hanley@mcgill.ca

For more information on migrant workers in Canada: www.migrantworker.ca
